# MRIgFUS in the treatment of spontaneous intracerebral hemorrhage

**DOI:** 10.1186/2050-5736-3-S1-O7

**Published:** 2015-06-30

**Authors:** Leodante da Costa, Nir Lipsman, Allison Bethune, Todd Mainprize, Kullervo Hynynen

**Affiliations:** 1 Sunnybrook Research Institute, Toronto, Ontario, Canada; 2 Sunnybrook Health Sciences Centre, Toronto, Ontario, Canada

## Background/introduction

Spontaneous cerebral hemorrhage (ICH) is a major cause of mortality and morbidity worldwide. Although the mechanisms leading to ICH are relatively well known, little improvement in outcomes has occurred over the years, in spite of significant advances in surgical techniques and medical management options. Evidence is available to suggest that liquefying and/or removing the clot after ICH might be beneficial. The objective of this work is to test the feasibility of use of MRI guided focused ultrasound (MRgFUS) in the treatment of ICH. Our hypotheses are that MRgFUS can be used safely to effectively cause clot lysis and it will provide good radiological resolution of ICH.

## Methods

At least 6 patients with a recent (< 72h) ICH and hematoma > 2 cm will be recruited. MRgFUS will be used to sonicate the clot leading to lysis. Except for the MRgFUS treatment, ICH patients will receive the same treatment as those not in the trial. Stereotactic aspiration can be added if deemed safe by the treating neurosurgeon. The primary outcome is feasibility (statistical analysis is not proposed); adverse events will be examined and analyzed. Secondary end point is the radiological progression of the clot.

## Results and conclusions

We expect that MRgFUS be feasible and safe, and clot size reduction will be seen in most patients. It’s possible that the procedure could have some therapeutic value for subjects with few or no other options for treatment.

